# Role of hyaluronan in regulating self-renewal and osteogenic differentiation of mesenchymal stromal cells and pre-osteoblasts

**DOI:** 10.1007/s00784-020-03259-8

**Published:** 2020-03-31

**Authors:** Maria B. Asparuhova, Vivianne Chappuis, Alexandra Stähli, Daniel Buser, Anton Sculean

**Affiliations:** 1 Laboratory of Oral Cell Biology, Dental Research Center, School of Dental Medicine, University of Bern, Freiburgstrasse 3, 3010 Bern, Switzerland; 2 Department of Oral Surgery and Stomatology, School of Dental Medicine, University of Bern, Freiburgstrasse 7, 3010 Bern, Switzerland; 3 Department of Periodontology, School of Dental Medicine, University of Bern, Freiburgstrasse 7, 3010 Bern, Switzerland

**Keywords:** Hyaluronic acid, Bone and soft tissue regeneration, Stemness, Growth factors, Extracellular matrix, Gene expression

## Abstract

**Objectives:**

The aim of the study was to investigate the impact of two hyaluronan (HA) formulations on the osteogenic potential of osteoblast precursors.

**Materials and methods:**

Proliferation rates of HA-treated mesenchymal stromal ST2 and pre-osteoblastic MC3T3-E1 cells were determined by 5-bromo-20-deoxyuridine (BrdU) assay. Expression of genes encoding osteogenic differentiation markers, critical growth, and stemness factors as well as activation of downstream signaling pathways in the HA-treated cells were analyzed by quantitative reverse transcription-polymerase chain reaction (qRT-PCR) and immunoblot techniques.

**Results:**

The investigated HAs strongly stimulated the growth of the osteoprogenitor lines and enhanced the expression of genes encoding bone matrix proteins. However, expression of late osteogenic differentiation markers was significantly inhibited, accompanied by decreased bone morphogenetic protein (BMP) signaling. The expression of genes encoding transforming growth factor-β1 (TGF-β1) and fibroblast growth factor-1 (FGF-1) as well as the phosphorylation of the downstream signaling molecules Smad2 and Erk1/2 were enhanced upon HA treatment. We observed significant upregulation of the transcription factor Sox2 and its direct transcription targets and critical stemness genes, Yap1 and Bmi1, in HA-treated cells. Moreover, prominent targets of the canonical Wnt signaling pathway showed reduced expression, whereas inhibitors of the pathway were considerably upregulated. We detected decrease of active β-catenin levels in HA-treated cells due to β-catenin being phosphorylated and, thus, targeted for degradation.

**Conclusions:**

HA strongly induces the growth of osteoprogenitors and maintains their stemness, thus potentially regulating the balance between self-renewal and differentiation during bone regeneration following reconstructive oral surgeries.

**Clinical relevance:**

Addition of HA to deficient bone or bony defects during implant or reconstructive periodontal surgeries may be a viable approach for expanding adult stem cells without losing their replicative and differentiation capabilities.

**Electronic supplementary material:**

The online version of this article (10.1007/s00784-020-03259-8) contains supplementary material, which is available to authorized users.

## Introduction

Due to its hygroscopic and viscoelastic properties as well as its high biocompatibility and non-immunogenic nature, hyaluronan (HA) has been utilized in various regenerative medical and tissue engineering applications [1]. HA is an anionic, non-sulfated glycosaminoglycan and a key component of the extracellular matrix (ECM) of vertebrate tissues. Contents of approximately 1–100 μg HA/g wet tissue weight were reported for most organs [2]. Measurements of HA content represent high interest since changes in HA content are often correlated with tissue remodeling and pathological processes [3]. HA is particularly prominent in non-mineralized periodontal tissues such as gingiva and periodontal ligament [4] compared to the lower quantities found in mineralized tissues such as cementum [5] and alveolar bone [6].

HA is involved in numerous biological processes related to tissue regeneration, such as regulation of cell adhesion, migration and proliferation, manipulation of cell differentiation, and mediation of cell signaling [7]. In addition, it exhibits anti-inflammatory [8], pro-angiogenic [9], and osteoinductive properties [10]. Although HA is a key component in the series of events associated with the wound healing process, i.e., inflammation, granulation tissue formation, epithelium formation, and tissue remodeling, detailed mechanisms of action remain largely uncovered and often controversial, especially in the healing of oral mineralized tissues following periodontal regenerative procedures and implant surgeries. It has been reported that the effect of HA on cellular proliferation and osteogenic differentiation in vitro largely depends on its molecular weight (MW) and concentration. Low MW HA (< 700 kDa) was mostly reported to stimulate cellular proliferation in calvaria- or tibia/femur condyle-derived mesenchymal cell cultures [11–13]. However, the effect of high MW HA (> 1000 kDa) on cellular proliferation is disputable. Some studies demonstrated that high MW HA promoted cellular adhesion and proliferation in a dose-dependent manner in rat calvarial mesenchymal [12] and human periodontal ligament [14] cell cultures, whereas others reported inhibition of cell growth in diverse cell types [11, 15, 16]. The effect of high MW HA on cellular differentiation is also open to question. High MW HA has been shown to significantly induce osteocalcin mRNA expression, mineralization, and alkaline phosphatase activity in rat calvarial-derived cell cultures, in a concentration-dependent manner [12]. In contrast, either no effect of high MW HA on mRNA expressions of bone-related genes in periodontal ligament cells [14] or even significant inhibition of the osteogenic differentiation of both mouse myoblastic and mouse mesenchymal cells, have been reported [16]. In vitro studies have shown that low MW HA exhibits osteogenic activity, both through the intramembranous and the endochondral paths of osteogenesis [11, 17].

In vivo, application of HA for bone regeneration has been demonstrated in craniofacial bone defects in various animal models [18]. To stimulate bone formation, HA is either (1) mixed with filler materials [9, 10, 19–21], (2) applied as a coating material [22], or (3) used as a carrier of growth factors and cells in the bone defect [23–25]. However, to date, only few clinical studies exist on the use of HA in reconstructive periodontal surgery [26–30]. Before conducting such clinical studies, a better understanding is needed of the influence of HA on the tissues comprising the periodontium, i.e., cementum, alveolar bone, periodontal ligament, and gingival connective tissue. In a recent study, we have demonstrated diverse positive effects of two commercially available HA formulations on primary human palatal and gingival fibroblasts, two cell types involved in soft tissue healing/regeneration following periodontal reconstructive therapies that utilize palatal connective tissue or free gingival grafts [31]. The observed pro-proliferative, pro-migratory, and pro-wound healing properties of the two HAs speak in favor of their clinical potential. Based upon these data, the goal of the present study was to investigate the effects of the two HAs on the growth of the mesenchymal stromal line, ST2 as well as the osteoprogenitor cell line, MC3T3-E1. The two commercially available HA preparations, planned to be used in reconstructive periodontal surgery, are considered high MW (≥ 1000 kDa) and appear close to the physiological HA found in many biological fluids and solid tissues [3]. The study further aimed to investigate the osteogenic response induced by the HAs in the two cell lines, thus providing insights into the mechanisms involved in HA-controlled osteogenesis and the clinical potential of the two HA formulations.

## Materials and methods

### Cell culture and HA preparations

Mesenchymal stromal ST2 cells (RIKEN; Tsukuba, Japan) and osteoblast progenitor MC3T3-E1 cells (ATCC, CRL-2593; Manassas, VA, USA) were grown in Dulbecco’s modified Eagle’s medium (DMEM) supplemented with 10% fetal bovine serum (FBS; Invitrogen, Zug, Switzerland). For differentiation experiments, media were supplemented with 50 μg/ml ascorbic acid (Invitrogen) and 2 mM β-glycerophosphate (Invitrogen) as described [32].

The investigated HA formulations were kindly provided by Regedent AG (Zurich, Switzerland) and previously described [31]. In brief, HA1 (hyaDENT) is a native non-crosslinked HA with MW of 2500 kDa, whereas HA2 (hyaDENT BG) is a formulation containing complexes of butanediol diglycidyl ether (BDDE)-crosslinked 1000 kDa-HA monomers and the above non-crosslinked form. Throughout the study, HA was used at a final concentration of 4 mg/ml in 0.3%FBS/DMEM as previously described [31]. The concentration was chosen based on (1) pilot experiments (data not shown) comparing 4 mg/ml-diluted HAs with the undiluted commercial preparations and (2) the assumption that each of the HA preparations will be naturally diluted by patients’ blood and saliva during the periodontal surgical procedure. In brief, for RNA analyses, cells were plated at 3 × 10^4^ cells/cm^2^ on HA-coated plates for 24 h. For protein analyses, 6 h after seeding at the same density, the adherent cells were starved in 0.3% FBS/DMEM for 18 h and then treated with HA for 30 min.

### Cell proliferation assay

Proliferation rates of HA-treated ST2 and MC3T3-E1 cells were determined using a 5-bromo-20-deoxyuridine (BrdU) chemiluminescent ELISA assay (Roche, Basel, Switzerland) as described [31, 33]. In brief, after 24 h of starvation, cells were plated in triplicate at 2 × 10^3^ cells/well in 3% FBS/DMEM on Black 96-well microtiter plates (Thermo Fisher Scientific, Reinach, Switzerland) coated with HA at a final concentration of 4 mg/ml. Cells were allowed to proliferate for 0, 24, 48, 72, and 96 h before labeling with BrdU for 2 h. BrdU uptake into newly synthesized DNA was determined according to manufacturer’s instructions using a luminometer Infinite® 200 (Tecan, Männedorf, Switzerland). Experimental values were normalized to the values of untreated control cells at the time point 0. Data represent means ± SD from three independent experiments.

### RNA analyses by qRT-PCR

Total RNA from HA-treated ST2 and MC3T3-E1 cells was isolated using the RNeasy Mini Kit (Qiagen, Basel, Switzerland). RNA, quantified on a NanoDrop 2000c (Thermo Fisher Scientific) instrument, was reverse transcribed, and relative transcripts for the osteogenic differentiation marker genes Col1a1, Col1a2, Spp1, Runx2, Bglap2, Ibsp, and Alpl, normalized to Gapdh, were measured using qRT-PCR as previously described [32]. Additionally, relative transcripts for Bmp2, Bmp4, Bmp7, Tgfb1, Fgf1, Sox2, Yap1, Bmi1, Ctgf, Ccnd1, Dkk1, Apc, and Gsk3b genes, normalized to Gapdh, were measured using FastStart Universal SYBR Green Master ROX (Roche) and the primer sequences listed in Electronic Supplementary Material, Table S1. qPCR was carried out in a 7500 Real-Time PCR System (Applied Biosystems, Rotkreuz, Switzerland) using a standard thermal cycling profile. Data were analyzed using the efficiency ∆∆Ct method [34]. All samples were run in duplicates. Data represent means ± SD from three independent experiments.

### Protein analyses by Western blot

Whole-cell extracts from HA-treated ST2 and MC3T3-E1 cells were prepared by lysis in radioimmunoprecipitation assay (RIPA) buffer as previously described [35]. Lysates were run on 10% sodium dodecyl sulfate-polyacrylamide gel electrophoresis (SDS-PAGE) and transferred to Amersham™ Protran® membrane (Sigma, Buchs, Switzerland). Visualization of proteins of interest was achieved by using anti-phospho-Smad1/5/8 (Cell Signaling Technology, Leiden, The Netherlands), anti-Smad1 (Cell Signaling Technology), anti-phospho-Smad2 (Thermo Fisher Scientific), anti-Smad2, anti-phospho-Erk1/2, anti-Erk, anti-active β-catenin, anti-phospho-β-catenin (all from Cell Signaling Technology), anti-vinculin (Sigma), and anti-GAPDH (abcam, Cambridge, UK) antibodies followed by horseradish peroxidase-conjugated secondary antibodies (MP Biomedicals, Santa Ana, CA, USA) for detection with the SuperSignal™ West Dura Substrate (Thermo Fisher Scientific). Immunoblot signals were analyzed by densitometry using ImageQuant Software (Molecular Dynamics, Groningen, the Netherlands). Data represent means ± SD from three independent experiments.

### Statistical analysis

All grouped data are means ± SD. Statistical analysis was completed using GraphPad InStat Software, version 3.05. Multiple comparisons were performed using one-way analysis of variance (ANOVA) with Tukey’s post-hoc test. Values of *P* < 0.05 were considered significant.

## Results

### The two HA preparations strongly stimulate the growth of ST2 and MC3T3-E1 cells and enhance the expression of genes encoding bone matrix proteins

We have previously shown that coating of cell culture plates with the non-crosslinked formulation of HA (HA1) resulted in the formation of a continuous uniform gel layer, whereas coating with the BDDE-crosslinked formulation of HA (HA2) resulted in the formation of HA meshes [31]. Thus, the two adherent cell lines ST2 and MC3T3-E1, both utilized in the study as a progenitor source of osteoblasts, were able to adhere on HA1-coated plates and their morphology did not differ from the morphology of untreated cells seeded on non-coated cell culture plastic (Electronic Supplementary Material, Fig. S1). In contrast, in the case of HA2, the two cell lines appeared to adhere solely on the cell culture plastic while HA2 was present in suspension (Electronic Supplementary Material, Fig. S1). Thus, due to the observed difference in the natural occurrence of the two HAs and as in our previously published research investigating the effects of the two HA formulations [31], a further distinction between HA applied as a coating or in suspension was not made and we refer to “HA treatment” throughout the present study.

**Fig. 1:**
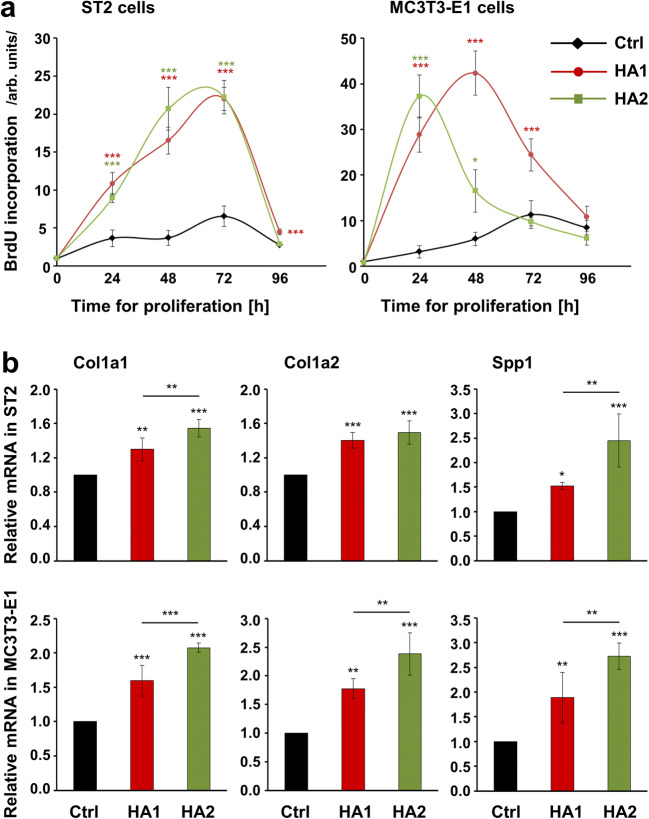
The two HA preparations strongly stimulate the growth of ST2 and MC3T3-E1 cells and enhance the expression of genes encoding bone matrix proteins. **a** Proliferation rates of HA-treated ST2 and MC3T3-E1 cells were assessed by BrdU incorporation into newly synthesized DNA immediately after plating (0 h) as well as at 24, 48, 72, and 96 h. Means ± SD from three independent experiments and significant differences to untreated control (Ctrl) cells at the time point 0; ***P < 0.001 and *P < 0.05 are shown. **b** Effect of HA1 and HA2 on Col1a1, Col1a2, and Spp1 mRNA levels in ST2 and MC3T3-E1 cells. Cells were treated with each of the two HA preparations for 24 h before total RNA was extracted and analyzed by qRT-PCR. Values normalized to Gapdh are expressed relative to the values of control (Ctrl) cells. Data represent means ± SD from three independent experiments. Significant differences to the respective control unless otherwise indicated, ***P < 0.001, **P < 0.01, *P < 0.05

We first assessed the effects of the two HA preparations on the proliferative rates of ST2 and MC3T3-E1 cells (Fig. 1a). Compared to untreated control cells, ST2 cells treated with either HA1 or HA2 showed a significant increase in BrdU uptake into newly synthesized DNA until they reached confluence approximately 65 h later (Fig. 1a, left panel). Interestingly, the proliferative rate of MC3T3-E1 cells treated with HA2 was greatly increased by 11.7-fold above the proliferative rate detected in control cells within the first 24 h (Fig. 1a, right panel, green line). An induction of 7.1-fold compared to control cells was observed in MC3T3-E1 cells treated with HA1 within 48 h (Fig. 1a, right panel, red line).

To assess the osteogenic potential of the two HA preparations, we monitored the expression of genes encoding bone matrix proteins such as collagen type I (Col1a1 and Col1a2) and osteopontin (also known as secreted phosphoprotein 1, Spp1) in HA-treated ST2 and MC3T3-E1 cells. Both HAs caused a significant increase in Col1a1, Col1a2, and Spp1 mRNAs above the expression levels detected in control cells (Fig. 1b). The crosslinked-HA2 formulation appeared more potent than the non-crosslinked HA1 and induced a significantly higher expression of the three bone matrix protein-coding genes in MC3T3-E1 cells as well as a higher expression of Col1a1 and Spp1 in ST2 cells.

The obtained data indicate a strong positive impact of the two HA formulations on the growth of ST2 and MC3T3-E1 cells. Furthermore, the data suggest a substantial positive impact of HA on the early stages of differentiation, namely matrix production. For both investigated functionalities, the crosslinked HA2 formulation appeared slightly more potent than the non-crosslinked HA1, especially in MC3T3-E1 pre-osteoblasts.

### The two HA preparations inhibit the expression of intermediate and late osteogenic differentiation markers as well as BMP-induced Smad signaling in ST2 and MC3T3-E1 cells

**Fig. 2:**
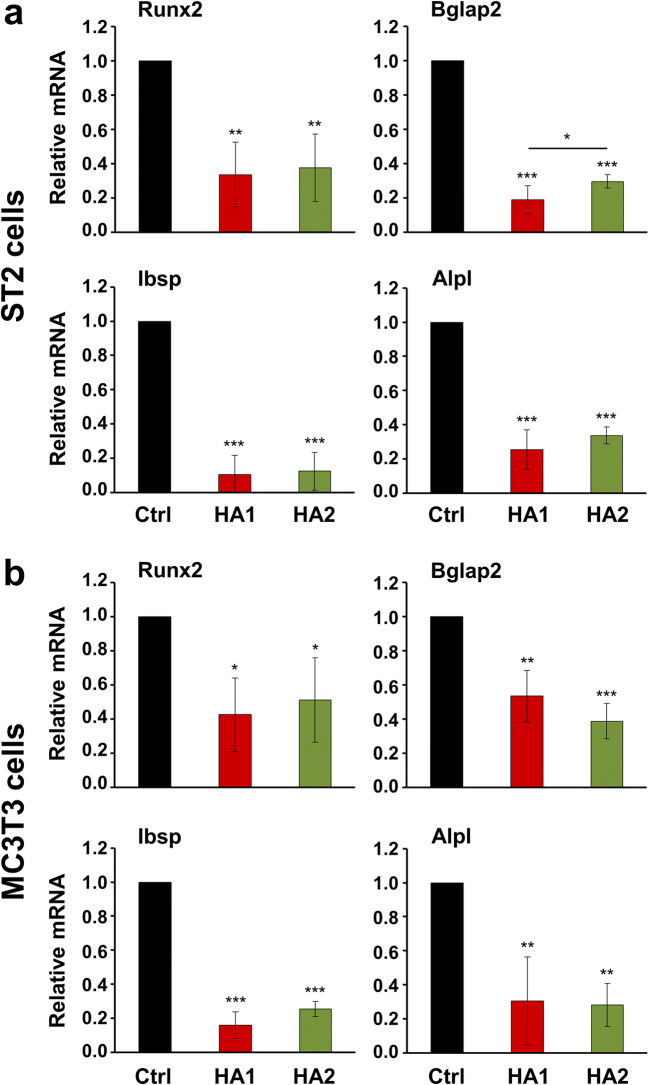
The two HA preparations inhibit the expression of intermediate and late osteogenic differentiation markers as well as BMP-induced Smad signaling in ST2 and MC3T3-E1 cells. **a**, **b** Effect of HA1 and HA2 on Runx2, Bglap2, Ibsp, and Alpl mRNA levels in ST2 (**a**) and MC3T3-E1 (**b**) cells. Each of the two cell lines was treated with HA1 or HA2 preparations for 24 h before total RNA was extracted and analyzed by qRT-PCR. Values normalized to Gapdh are expressed relative to the values of untreated control (Ctrl) cells. Data represent means ± SD from three independent experiments. Significant differences to the respective control unless otherwise indicated, ***P < 0.001, **P < 0.01, *P < 0.05

To further assess the osteogenic potential of HA, we monitored the expression of genes encoding intermediate and late differentiation markers such as runt-related transcription factor 2 (Runx2), osteocalcin (or bone gamma-carboxyglutamate protein 2, Bglap2), bone sialoprotein (or integrin-binding sialoprotein, Ibsp), and alkaline phosphatase (Alpl) in HA-treated ST2 and MC3T3-E1 cells. Both HA preparations caused a strong and significant inhibition of all four differentiation markers in both progenitor cell lines compared to control cells (Fig. 2a, b). In ST2 cells, HA1 appeared to be slightly but significantly more potent (by 1.6-fold, *P* < 0.05) than HA2 in suppressing Bglap2 expression (Fig. 2a). In agreement with the gene expression analysis, the mineral deposition capacity of HA-treated ST2 and MC3T3-E1 cells was strongly reduced as detected by alizarin red stain (Electronic Supplementary Material, Fig. S2). The obtained data strongly suggest an inhibited progression of HA-treated cells to the next stages of osteoblast differentiation, namely maturation of the ECM with alkaline phosphatase and subsequent matrix mineralization.

**Fig. 3:**
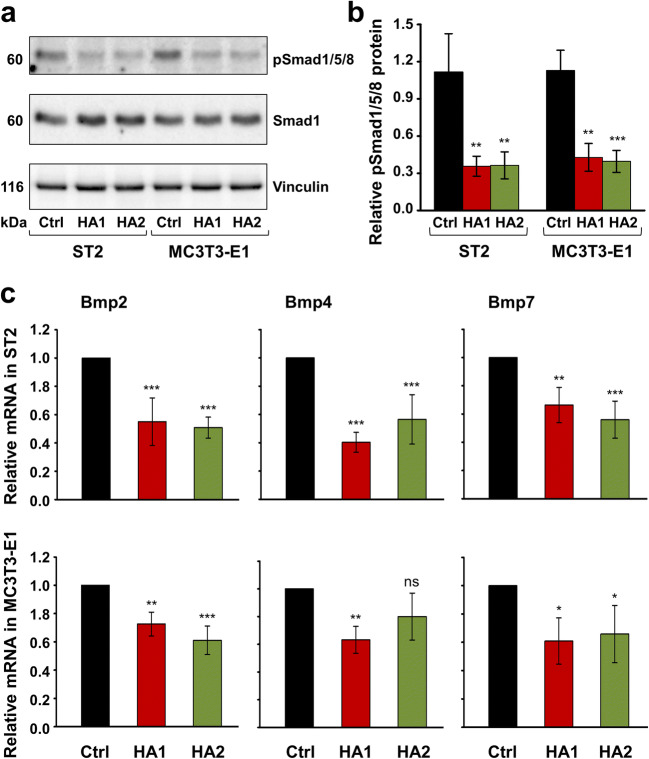
The two HA preparations inhibit BMP-activated Smad signaling in ST2 and MC3T3-E1 cells. **a**, **b** Immunoblot analysis of phospho-Smad1/5/8 (pSmad1/5/8) protein (**a**) in whole-cell extracts from ST2 and MC3T3-E1 cells treated with each of the two HA preparations. Blots for total Smad1 protein as well as the vinculin loading control are also shown **a**. The bar chart (**b**) represents densitometric quantification of the immunoblots. pSmad1/5/8 levels are normalized to the total Smad1 protein used as internal control. Data represent means ± SD from three independent experiments. Significant differences to the respective control (Ctrl) cells of each of the two cell lines, ***P < 0.001, **P < 0.01. **c** Effect of HA1 and HA2 on Bmp2, Bmp4, and Bmp7 mRNA levels in ST2 and MC3T3-E1 cells. Cells were treated with each of the two HA preparations for 24 h before total RNA was extracted and analyzed by qRT-PCR. Values normalized to Gapdh are expressed relative to the values of control (Ctrl) cells. Data represent means ± SD from three independent experiments. Significant differences to the respective control, ***P < 0.001, **P < 0.01, *P < 0.05, ns = not significant

In agreement with these data, we observed strong downregulation of Smad1/5/8 signaling in HA-treated ST2 and MC3T3-E1 cells (Fig. 3a, b). Compared to the basal levels of phospho-Smad1/5/8 detected in control cells of the two progenitor lines, the phosphorylation of Smad1/5/8 protein significantly decreased by approximately 3-fold in cells treated with each of the two HA preparations (Fig. 3b).

Smad1, 5, and 8 are receptor-regulated transcription factors (R-Smads) activated by BMPs [36]. BMPs induce Runx2 expression in mesenchymal progenitor cells namely through the action of R-Smads [37], and R-Smads in turn interact with Runx2 to regulate the transcription of target genes leading to osteoblast differentiation [38]. HA-treated ST2 and MC3T3-E1 cells were further characterized by decreased expression of Bmp2, 4, and 7 genes, encoding three osteogenic BMPs (Fig. 3c). Taken together, our data indicate that the two HA preparations negatively regulate the BMP-Runx2 axis involved in osteogenic differentiation.

### The two HA preparations significantly enhance the expression of genes encoding TGF-β1 and FGF-1 as well as the phosphorylation of Smad2 and Erk1/2 signaling molecules in ST2 and MC3T3-E1 cells

**Fig. 4:**
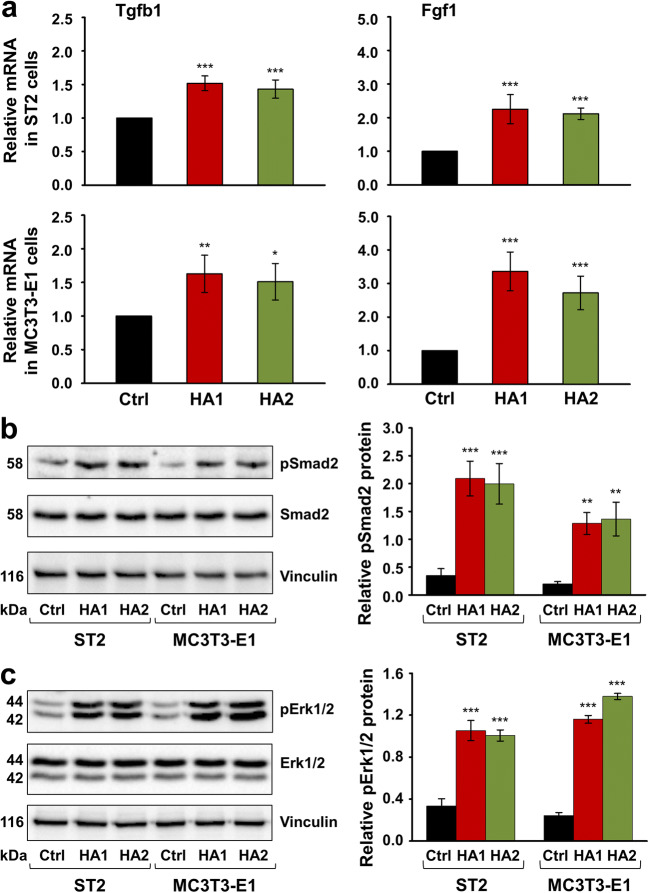
The two HA preparations significantly enhance the expression of genes encoding TGF-β1 and FGF-1 growth factors as well as the phosphorylation of Smad2 and Erk1/2 signaling molecules in ST2 and MC3T3-E1 cells. **a** Effect of HA1 and HA2 on Tgfb1 and Fgf1 mRNA levels in ST2 and MC3T3-E1 cells. Cells of each of the two lines were treated with each of the two HA preparations for 24 h before total RNA was extracted and analyzed by qRT-PCR. Values normalized to Gapdh are expressed relative to the values of control (Ctrl) cells. Data represent means ± SD from three independent experiments. Significant differences to the respective control, ***P < 0.001, **P < 0.01, *P < 0.05. **b**, **c** Immunoblot analyses of phospho-Smad2 (pSmad2) (**b**) and phospho-Erk1/2 (pErk1/2) (**c**) proteins in whole-cell extracts from HA-treated ST2 and MC3T3-E1 cells. Blots for total Smad2 and Erk1/2 proteins as well as the vinculin loading control are also shown. The bar charts represent densitometric quantifications of the immunoblots. pSmad2 and pErk1/2 levels are normalized to the respective total proteins used as internal controls. Data represent means ± SD from three independent experiments. Significant differences to the respective control (Ctrl) cells of each of the two cell lines, ***P < 0.001, **P < 0.01

Several lines of evidence indicate that TGF-β1 and FGFs regulate proliferation and differentiation in osteoblasts [39, 40]. To investigate how HA increases the proliferation and bone matrix production in ST2 and MC3T3-E1 progenitor cells but blocks their differentiation into mature osteoblasts, we tested the expression of genes encoding TGF-β1 and FGF-1 in HA-treated cells of the two lines. We chose FGF-1 as a ligand that binds all FGF receptor isoforms [41]. qRT-PCR analyses revealed a significant induction of both growth factors in ST2 and MC3T3-E1 cells exposed to each of the two HAs compared to basal expression levels detected in control cells (Fig. 4a). No trend for a difference in efficacy between the two HA formulations was observed.

To gain insights into the mechanisms whereby the two HAs exert their effects on growth factor gene expression in the osteoblast precursor cell lines, we investigated the activation state of two signaling molecules, Smad2 and Erk1/2 by immunoblotting. Smad2 responds to TGF-β1 [36], whereas FGFs often signal through the mitogen-activated protein kinase (MAPK) Erk1/2 [42]. Compared to the basal levels of phospho-Smad2 and phospho-Erk1/2 detected in control cells, phosphorylation of each of the two proteins significantly increased in both ST2 and MC3T3-E1cells treated with either HA formulation for 30 min (Fig. 4b, c). In particular, the phosphorylation of the TGF-β1-specific Smad2 in HA-treated cells was upregulated between 5.8- and 7.0-fold compared to control cells (Fig. 4b), whereas the phosphorylation of Erk1/2 increased by 3.0–5.7-fold (Fig. 4c).

### The two HA preparations induce expression of the transcription factor Sox2 and its direct targets Yap1 and Bmi1 in ST2 and MC3T3-E1 cells

**Fig. 5:**
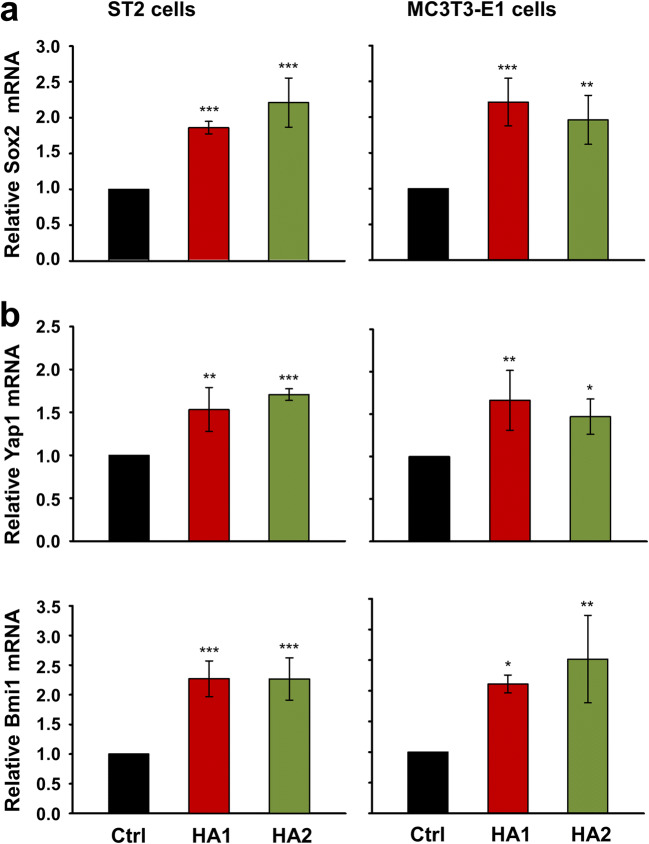
The two HA preparations induce expression of the transcription factor Sox2 and its direct targets Yap1 and Bmi1 in ST2 and MC3T3-E1 cells. **a**, **b** Effect of HA1 and HA2 on Sox2 (**a**), Yap1, and Bmi1 (**b**) mRNA levels in ST2 and MC3T3-E1 cells. Cells were treated with each of the two HA preparations for 24 h before total RNA was extracted and analyzed by qRT-PCR. Values normalized to Gapdh are expressed relative to the values of control (Ctrl) cells. Data represent means ± SD from three independent experiments. Significant differences to the respective control, ***P < 0.001, **P < 0.01, *P < 0.05

Earlier studies have established a mechanistic link between expression of the transcription factor Sox2 and FGF or TGF-β signaling in osteoblasts or cancer cells, respectively [43–45]. Furthermore, Sox2 has been described as a negative regulator of osteoblast differentiation and maturation in vivo [46, 47]. Thus, to further dissect the mechanism underlying the response of mesenchymal stromal and pre-osteoblastic cells to HA, we tested the expression of Sox2 in HA-treated ST2 and MC3T3-E1 cells, respectively. Compared to control cells, either HA preparation upregulated the expression levels of Sox2 in both cell lines (Fig. 5a).

Sox2, as a member of the high mobility group (HMO) domain Sox family of transcription factors, is required to maintain the stemness and self-renewal of embryonic [48] as well as adult stem cells [49–51]. This prompted us to investigate the influence of HA on the expression levels of two critical stemness genes, Yap1 [52] and Bmi1 [53], which were shown to be direct transcriptional targets of Sox2 in the osteoblast lineage [49, 50]. qRT-PCR analyses revealed that treatment of the two progenitor cell lines with HA resulted in increased Yap1 and Bmi1 mRNA levels above the levels obtained in control cells (Fig. 5b). No trend for a more pronounced effect of one or the other HA on the induction of these two genes was observed.

Taken together, our data strongly suggest that the two HA preparations maintain the self-renewal (stemness) potential of mesenchymal stromal ST2 and pre-osteoblastic MC3T3-E1 cells.

### The two HA preparations inhibit Wnt signaling in ST2 and MC3T3-E1 cells

**Fig. 6:**
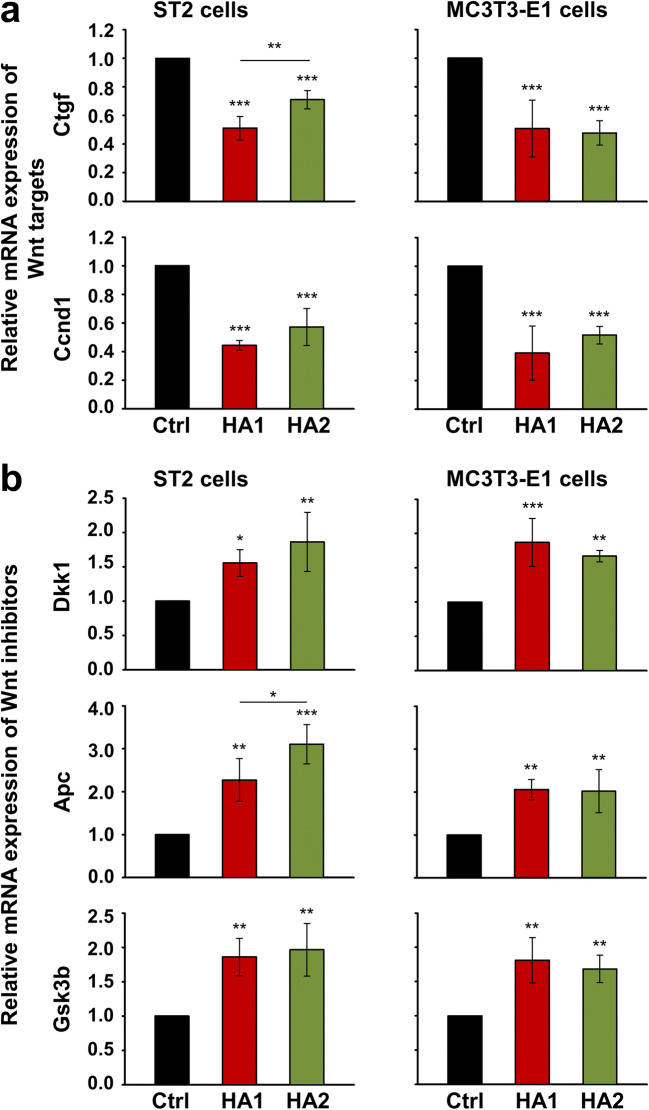
The two HA preparations influence the expression of genes involved in the Wnt signaling pathway in ST2 and MC3T3-E1 cells. **a** HA downregulates the mRNA expression levels of two Wnt target genes, Ctgf and Ccnd1, encoding connective tissue growth factor and cyclin D1 protein, respectively. ST2 and MC3T3-E1 cells were treated with each of the two HA preparations for 24 h before total RNA was extracted and analyzed by qRT-PCR. Values normalized to Gapdh are expressed relative to the values of control (Ctrl) cells. Data represent means ± SD from three independent experiments. Significant differences to the respective control unless otherwise indicated, ***P < 0.001, **P < 0.01. **b** HA upregulates the mRNA expression levels of three Wnt inhibitors, Dkk1, Apc, and Gsk3b, encoding the dickkopf-related protein 1, adenomatous polyposis coli, and glycogen synthase kinase 3 beta protein, respectively. Cells were treated as in **a** before mRNA levels of the respective genes were analyzed by qRT-PCR. Values normalized to Gapdh are expressed relative to the values of control (Ctrl) cells. Data represent means ± SD from three independent experiments. Significant differences to the respective control unless otherwise indicated, ***P < 0.001, **P < 0.01, *P < 0.05

The canonical Wnt signaling pathway promotes differentiation in osteoprogenitor cells [54, 55]. Furthermore, the inhibitory effect of Sox2 on the osteogenic differentiation has been partially explained by interfering with Wnt signaling [43, 56]. Thus, inhibited Wnt signaling could potentially provide a mechanistic explanation for the inhibited expression of osteogenic differentiation markers in HA-treated ST2 and MC3T3-E1 cells. We first tested the influence of HA on the expression of two prominent Wnt target genes, Ctgf and Ccnd1, encoding connective tissue growth factor and cyclin D1 protein, respectively. Our results showed a very significant (*P* < 0.001) downregulation of the two genes upon HA treatment of the two cell lines compared to control cells (Fig. 6a). In contrast, negative Wnt regulators such as Dkk1, Apc, and Gsk3b, encoding the dickkopf-related protein 1, adenomatous polyposis coli, and glycogen synthase kinase 3 beta, respectively, were significantly upregulated by each of the two HAs compared to their basal expression levels in control cells (Fig. 6b). In isolated cases, one HA formulation appeared to be more potent than the other, e.g., HA1 caused a significantly stronger downregulation of Ctgf mRNA (Fig. 6a, upper left panel), whereas HA2 more strongly induced Apc expression in ST2 cells (Fig. 6b, middle left panel).

**Fig. 7:**
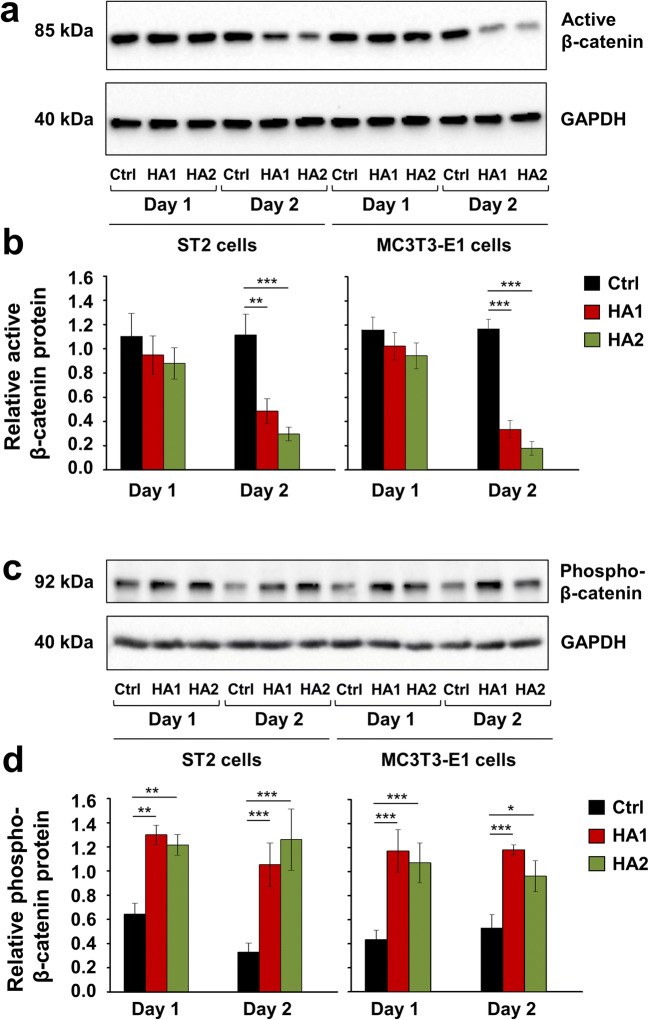
The two HA preparations inhibit Wnt signaling in ST2 and MC3T3-E1 cells. **a**–**d** Immunoblots of active β-catenin (**a**, **b**) and phospho-β-catenin (**c**, **d**) proteins in whole-cell extracts of ST2 and MC3T3-E1 cells treated with each of the two HA preparations. Cell lysates were collected on two consecutive days after the treatment. Anti-GAPDH served as loading control. Densitometric analyses (**b**, **d**) of the immunoblots shown in **a** and **c**. Active β-catenin (**a**, **b**) and phospho-β-catenin (**c**, **d**) protein levels are normalized to the GAPDH loading controls. Means ± SD from three independent experiments and significant differences to the respective control (Ctrl) cells, ***P < 0.001, **P < 0.01, *P < 0.05 are shown. No statistically significant differences between identically treated cells on days 1 and 2 after the stimulation were detected

To gain further insight into the mechanism determining the inhibitory effect of HA on the Wnt signaling and osteoblast differentiation of ST2 and MC3T3-E1 cells, we monitored the levels of active non-phosphorylated as well as phosphorylated β-catenin protein on two consecutive days after HA treatment (Fig. 7). In the absence of a Wnt stimulus, β-catenin is constitutively phosphorylated by GSK3β and then targeted for degradation by ubiquitination [57]. On the contrary, when GSK3β activity is inhibited by Wnt, non-phosphorylated β-catenin accumulates in the cell cytoplasm followed by its translocation into the nucleus [58]. There, β-catenin acts as a transcriptional coactivator for a family of transcription factors, which are otherwise bound to DNA in a repressive complex not permitting the expression of Wnt target genes [59]. Short treatment with each of the two HA preparations resulted in a slight but not significant decrease in the levels of active β-catenin protein in each of the two cell lines after 24 h. However, on day 2 after the treatment, active β-catenin levels were strongly reduced by 2.3–3.8- and 3.5–6.5-fold in HA-treated ST2 and MC3T3-E1 cells, respectively, compared to the respective control cells (Fig. 7a, b). In contrast, β-catenin phosphorylation was significantly enhanced by the HA preparations in each of the two cell lines on day 1 and remained induced on day 2 after the treatment (Fig. 7c, d), suggesting a role of HA in targeting β-catenin for degradation.

These results indicate that along with the described downregulation of BMP-induced Smad signaling, the two HA preparations cause inhibition of Wnt signaling, which ultimately contributes to the prominent role of HA in maintaining the self-renewal capabilities of mesenchymal stromal and osteoprogenitor cells.

## Discussion

HA is a key structural element in both mineralized and non-mineralized periodontal tissues, such as alveolar bone and cementum [5, 6], gingiva, and periodontal ligament [4]. The unique hygroscopic and viscoelastic properties of HA contribute to the series of events associated with the soft tissue wound healing process in periodontal regeneration [60]. Based on the fact that wound healing in different tissues follow similar biological principles, it is conceivable that HA has comparable roles in the healing of the mineralized tissues of the periodontium. In a recent study, we investigated the role of two HA formulations on the cellular behavior and wound healing properties of primary human palatal and gingival fibroblasts as the two major cell types involved in soft tissue healing/regeneration following periodontal reconstructive therapies [31]. Bone marrow–derived mesenchymal stromal cells as well as calvaria-derived pre-osteoblasts are widely used to study osteogenic differentiation in vitro and to gain an insight into the mechanisms driving the bone healing process. Thus, the objective of the present study was to evaluate the effects of the two HA preparations on the osteogenic potential of ST2 mesenchymal stromal and MC3T3-E1 pre-osteoblastic cell lines.

**Fig. 8:**
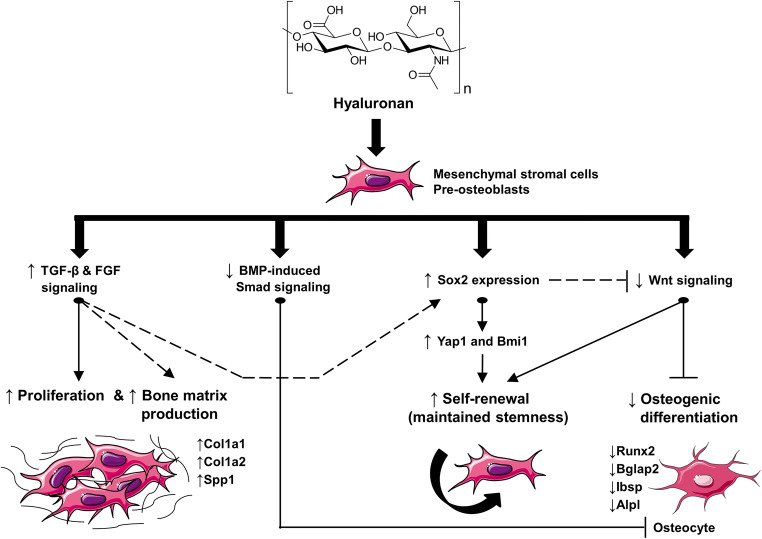
Model for the effects of the two investigated HA formulations on the behavior and functionality of mesenchymal stromal ST2 and pre-osteoblastic MC3T3-E1 cells. Treatment of each of the two cell lines, used as a progenitor source of osteoblasts, with HA (a structural formula is presented) results in induced cellular proliferation and bone matrix production, as suggested by the upregulated expression of Col1a1, Col1a2, and Spp1 genes encoding bone matrix proteins. These effects are accompanied and likely influenced by induced TGF-β and FGF signaling in the HA-treated cells. Signaling through both BMP-induced Smad and Wnt pathways is strongly downregulated upon HA treatment, which results in inhibited progression of the osteogenic differentiation as evidenced by decreased expression of intermediate and late osteogenic differentiation marker genes such as Runx2, Bglap2, Ibsp, and Alpl. Finally, HA maintains the stemness of osteoprogenitors by inducing the expression of the transcription factor Sox2 and its direct targets Yap1 and Bmi1. Inhibited Wnt signaling and induced TGF-β/FGF signaling may contribute to the self-renewal capability of HA-treated osteoprogenitors. Taken together, our results suggest a prominent role of HA in inducing the growth, and maintaining the stemness and differentiation potential of mesenchymal stromal and pre-osteoblastic cells. Solid lines in the model represent direct evidence and common knowledge; dashed lines display links that are solidly supported by literature reports. For more details on the displayed links between the HA-induced functionalities, please refer to the text

Our data, summarized in Fig. 8, demonstrated that the two HAs exhibit multiple actions on the functionalities of the two cell lines. First, we could show that HA strongly stimulates the proliferation of the two progenitor lines and enhances the expression of genes encoding proteins typical for yet uncalcified bone matrix, suggesting a potent role of HA in increasing the pool of committed osteoblasts and in triggering the early stages of the differentiation, namely bone matrix production. Second, we demonstrated a prominent role of HA in maintaining the stemness of mesenchymal stromal and pre-osteoblastic cells by acting on several different levels: (1) downregulation of BMP-induced Smad signaling, (2) induction of the expression of Sox2 and its downstream targets Yap1 and Bmi1 as critical stemness genes, (3) inhibition of Wnt signaling, and (4) suppression of genes typical for mineralized bone matrix. The multiple effects of the investigated HAs on the behavior and function of the two progenitor cell lines could be considered as either independent or strongly interrelated (potential interrelations between the HA-induced effects described in the present study are displayed in Fig. 8).

Controversial effects of HA on both cellular proliferation and osteogenic differentiation in vitro have been reported in the literature [11–15, 17, 61]. The nature of the observed effects is tightly related to the cellular context, concentration and MW of HA, and mode of delivery of HA to the cell culture [18]. Here, we reported a pro-proliferative effect of HAs with a MW close to that of the physiological HA present in healthy solid tissues (1000–6000 kDa) [3]. In our experiments, the HA preparations were applied to cell cultures diluted by 3.5–4.5-fold, thus considering their potential dilution by patient’s blood and saliva at the defect side. Moreover, the positive effects on the cellular proliferation have been observed for both the non-crosslinked HA1 and the crosslinked HA2 formulation. The pro-proliferative effects of the investigated HAs could be mechanistically explained by the increased expression of genes encoding TGF-β1 and FGF-1 growth factors as well as the induction of Smad2 and Erk1/2 signaling in the HA-treated ST2 and MC3T3-E1 cells. TGF-β1 and FGF signaling pathways have been reported to induce cellular proliferation in the osteoblast lineage [40, 62]. Furthermore, TGF-β1 has been recognized as a key promoter of collagen expression [63] and early stages of bone formation, namely bone matrix production [32, 39]. FGFs, in their turn, were shown to prominently induce the osteopontin-encoding Spp1 gene in various cell types [64–66], but they negatively influenced the expression of Col1 [67]. This suggests that TGF-β1 and FGF-1 might not be the only factors induced by HA that could explain the upregulation of Spp1 and Col1 genes in the HA-treated osteoprogenitors. The same is valid for the observed suppression of intermediate and late osteogenic differentiation markers upon HA treatment, which might not solely result by the downregulation of BMP-induced Smad phosphorylation or inhibition of Wnt signaling. Inhibited osteogenesis upon HA treatment might be an end point achieved by several mechanisms, e.g., we cannot exclude a negative effect of HA on the insulin-like growth factor (IGF) signaling pathway that is known to induce osteoblast differentiation [68]. A number of animal and human genetics studies have identified a role for Wnt signaling in promoting osteoblast function and bone formation, and Wnt signals often cooperate with BMPs to induce osteoblast differentiation [69]. Thus, the downregulation of Wnt signaling observed in our study is an important mechanism by which HA inhibits late osteogenic differentiation marker expression.

Consequently, we showed that HA induces the expression of the transcription factor Sox2 and its direct targets Yap1 and Bmi1. Yap1 (Yes-associated protein 1) is a transcriptional coactivator of the TEAD transcription factors, and Bmi1 is a polycomb complex transcriptional repressor. All three genes are implicated in maintaining pluripotency and self-renewal in embryonic [48, 52, 53] as well as in adult stem cells [49–51], suggesting a prominent role of HA in maintaining the stemness and differentiation potential of mesenchymal stromal and pre-osteoblastic cells. Our in vitro study has certain limitations, e.g., further research is needed to elucidate if HA may control cell fate decisions during bone formation in vivo. It may be speculated that HA provides the time for progenitor cells to proliferate and reach a critical cell mass before differentiation is initiated. Differentiation can then be triggered by other factors at the defect side, e.g., by signals coming from autologous bone used as a bone graft material [32]. It has already been shown that autologous bone combined with an esterified HA preparation accelerated new bone formation after surgical treatment of infrabony defects [26].

In conclusion, our data strongly suggest that addition of HA to deficient bone or bony defects during implant or reconstructive periodontal surgeries might be a useful approach for expanding adult stem cells without losing their replicative and differentiation capabilities. The ability of the two investigated HAs to expand the pool of osteoprogenitors and to maintain their stemness could be combined with the possibility for triggering osteogenic differentiation either by manipulation of HA concentration and/or MW or by combination of HA with autologous bone or another osteoinductive bone substitute material, thus providing a complete solution for bone regeneration during reconstructive periodontal and implant surgeries.

## Electronic supplementary material

ESM 1 (PDF 621 kb)

## Data Availability

All data generated and analyzed during this study are included in this article and its Electronic Supplementary Material file.
