# Supplementary material for: Role of hyaluronan in regulating self-renewal and osteogenic differentiation of mesenchymal stromal cells and pre-osteoblasts
Source: Clin Oral Investig. 2020 Mar 31;24(11):3923–37. doi: 10.1007/s00784-020-03259-8 (PMC7544712; doi:10.1007/s00784-020-03259-8)
Supplement: Supplementary file 1 — (PDF 621 kb) [file 784_2020_3259_MOESM1_ESM.pdf]

Electronic Supplementary Material for:

Role of hyaluronan in regulating self-renewal and osteogenic differentiation of mesenchymal stromal cells and pre-osteoblasts

Maria B. Asparuhova[1,2,3*], Vivianne Chappuis[2], Alexandra Stähli[3], Daniel Buser[2], Anton Sculean[3]

[1]Laboratory of Oral Cell Biology, Dental Research Center, School of Dental Medicine, University of Bern, Freiburgstrasse 3, 3010 Bern, Switzerland

[2]Department of Oral Surgery and Stomatology, School of Dental Medicine, University of Bern, Freiburgstrasse 7, 3010 Bern, Switzerland

[3]Department of Periodontology, School of Dental Medicine, University of Bern, Freiburgstrasse 7, 3010 Bern, Switzerland

***Corresponding author:** Maria B. Asparuhova
Laboratory of Oral Cell Biology
School of Dental Medicine, University of Bern
Dental Research Center, a platform of sitem-insel
Freiburgstrasse 3, CH-3010 Bern, Switzerland
Tel: +41 31 632 86 22
Email: mariya.asparuhova@zmk.unibe.ch

Content:

Figure S1. Morphology of HA–treated ST2 and MC3T3-E1 cells.

Figure S2. Mineral deposition capacity of HA–treated ST2 and MC3T3-E1 cells assessed by Alizarin red stain.

Table S1. Primer sequences.

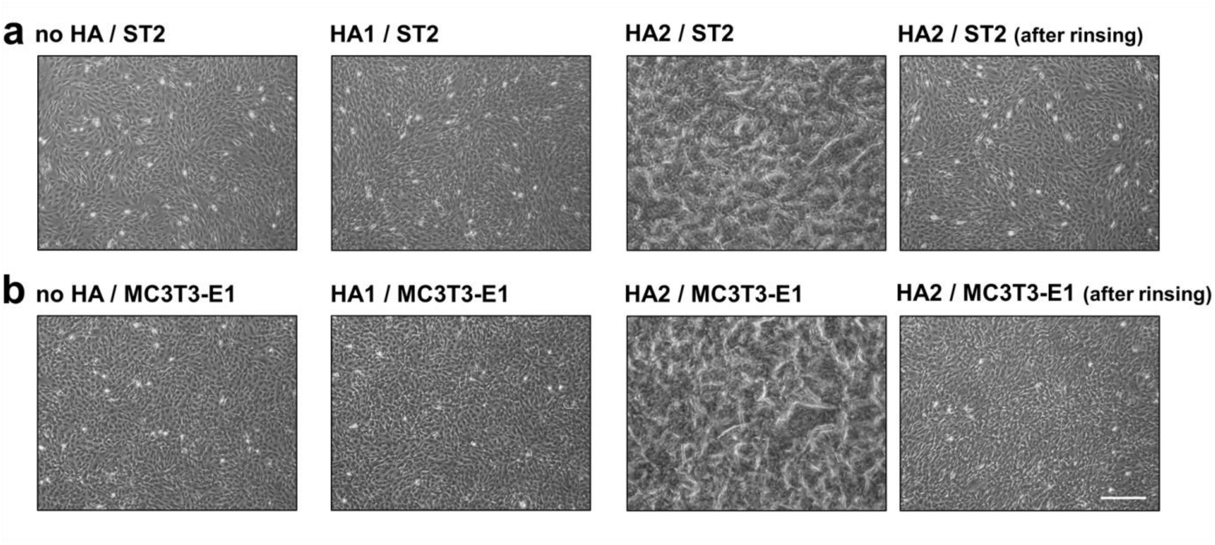

Fig. S1 Morphology of HA–treated ST2 and MC3T3-E1 cells. Images of ST2 **(a)** and MC3T3-E1 **(b)** cells seeded on uncoated (left panels, no HA), HA1- or HA2-coated (middle panels) wells. Cells were plated at 3 x 10^4 cells/cm^2 for 24 h before images were taken on a Leica DM IL LED microscope equipped with Leica DFC420 C camera. Both cell types, ST2 and MC3T3-E1, were able to adhere on HA1-coated plates and their morphology did not differ from the morphology of untreated cells seeded on non-coated cell culture plastic (**a** and **b**, compare left with middle panels). In contrast, on HA2-coated plates, cells appeared to adhere solely on the cell culture plastic while HA2 was present in suspension. Images of ST2 and MC3T3-E1 cells plated on HA2-coated wells after rinsing with PBS are shown (**a** and **b**, right panels). Bar, 500 µm

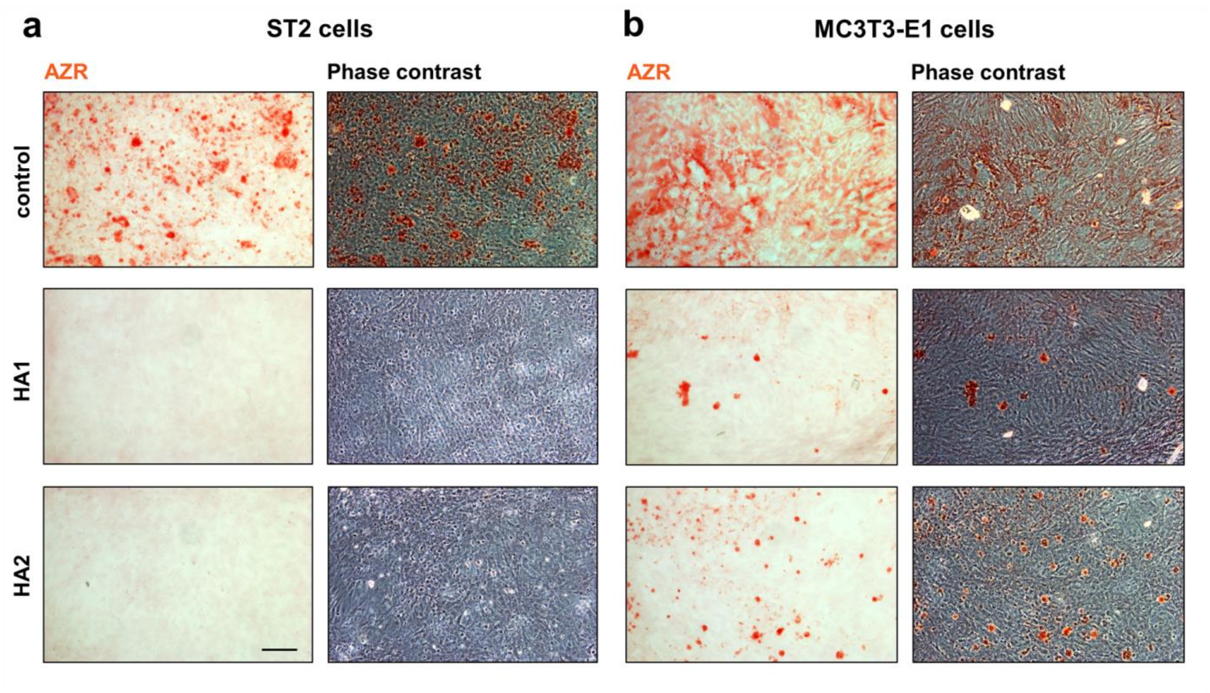

Fig. S2 Mineral deposition capacity of HA–treated ST2 (a) and MC3T3-E1 (b) cells assessed by Alizarin red stain. Cells were seeded on uncoated (control), HA1- or HA2-coated wells of 8-well chamber slides (ThermoFisher Scientific, Reinach, Switzerland) in osteogenic media at a density of 3 x 10^4 cells/cm^2. After 21 days, cells were fixed in 96 % ethanol for 15 min and stained with 0.2 % Alizarin Red S (AZR)(Sigma-Aldrich, Basel, Switzerland), pH 6.4 at room temperature for 1 h. Images (including phase contrast) were acquired on an Olympus BX-51. Representative images of the staining in each of the experimental groups are shown. Scale bar, 500 µm.

Table S1 Primer Sequences

Gene symbol	Gene bank accession number	Primer pair (fwd/rev)	Amplicon size (bp)
Bmp2	NM_007553	5'-GGGACCCGCTGTCTTCTAGT-3'<br>5'-TCAACTCAAATTCGCTGAGGAC-3'	154
Bmp4	NM_007554	5'-TTCCTGGTAACCGAATGCTGA-3'<br>5'-CCTGAATCTCGGCGACTTTTT-3'	114
Bmp7	NM_007557	5'-CCTGTCCATCTTAGGGTTGCC-3'<br>5'-GGCCTTGTAGGGGTAGGAGA-3'	151
Tgfb1	NM_011577	5'-CTTCAATACGTCAGACATTCGGG-3'<br>5'-GTAACGCCAGGAATTGTTGCTA-3'	142
Fgf1	NM_010197	5'-CCCTGACCGAGAGGTTCAAC-3'<br>5'-GTCCCTTGTCCCATCCACG-3'	122
Sox2	NM_011443	5'-CCCACCTACAGCATGTCCTAC-3'<br>5'-GCCTCGGACTTGACCACAG-3'	83
Yap1	NM_001171147	5'-TACTGATGCAGGTACTGCGG-3'<br>5'-TCAGGGATCTCAAAGGAGGAC-3'	180
Bmi1	NM_007552	5'-ATCCCCACTTAATGTGTGTCCT-3'<br>5'-CTTGCTGGTCTCCAAGTAACG-3'	116
Ctgf	NM_010217	5'-GGGCCTCTTCTGCGATTTC-3'<br>5'-ATCCAGGCAAGTGCATTGGTA-3'	151
Ccnd1	NM_007631.2	5'-AGAAGTGCGAAGAGGAGGTC-3'<br>5'-AGTTCCATTTGCAGCAGCTC-3'	213
Dkk1	NM_010051	5'-CAGTGCCACCTTGAACTCAGT-3'<br>5'-CCGCCCTCATAGAGAACTCC-3'	129
Apc	NM_007462	5'-CCCCGGAGTGAAACTACGC-3'<br>5'- GGGGACAGGACTGCATTCTC-3'	97
Gsk3b	NM_019827	5′-TGGCAGCAAGGTAACCACAG-3′<br>5′-CGGTTCTTAAATCGCTTGTCCTG-3′	189
